# Non-pneumococcal mitis-group streptococci confound detection of pneumococcal capsular serotype-specific loci in upper respiratory tract

**DOI:** 10.7717/peerj.97

**Published:** 2013-06-25

**Authors:** Maria da Gloria Carvalho, Fabiana C. Pimenta, Iaci Moura, Alexis Roundtree, Robert E. Gertz, Zhongya Li, Geofrey Jagero, Godfrey Bigogo, Muthoni Junghae, Laura Conklin, Daniel R. Feikin, Robert F. Breiman, Cynthia G. Whitney, Bernard W. Beall

**Affiliations:** 1 National Center for Immunization and Respiratory Diseases, Centers for Disease Control and Prevention, Atlanta, USA; 2 Kenya Medical Research Institute, Kenya; 3 International Emerging Infections Program, Centers for Disease Control and Prevention, Kenya

**Keywords:** Pneumococcal serotype-specific loci, Mitis group streptococci, Oropharyngeal and nasopharyngeal flora, PCR for serotype deduction

## Abstract

We performed culture-based and PCR-based tests for pneumococcal identification and serotyping from carriage specimens collected in rural and urban Kenya. Nasopharyngeal specimens from 237 healthy children <5 years old (C-NPs) and combined nasopharyngeal/oropharyngeal specimens from 158 adults (A-NP/OPs, 118 HIV-positive) were assessed using pneumococcal isolation (following broth culture enrichment) with Quellung-based serotyping, real-time *lyt*A-PCR, and conventional multiplexed PCR-serotyping (cmPCR). Culture-based testing from C-NPs, HIV-positive A-NP/OPs, and HIV-negative A-NP/OPs revealed 85.2%, 40.7%, and 12.5% pneumococcal carriage, respectively. In contrast, cmPCR serotypes were found in 93.2%, 98.3%, and 95.0% of these sets, respectively. Two of 16 *lyt*A-negative C-NPs and 26 of 28 *lyt*A-negative A-NP/OPs were cmPCR-positive for 1–10 serotypes (sts) or serogroups (sgs). A-NP/OPs averaged 5.5 cmPCR serotypes/serogroups (5.2 in HIV-positive, 7.1 in HIV-negative) and C-NPs averaged 1.5 cmPCR serotypes/serogroups. cmPCR serotypes/serogroups from *lyt*A-negative A-NP/OPs included st2, st4, sg7F/7A, sg9N/9L, st10A, sg10F/10C/33C, st13, st17F, sg18C/18A/18B/18F, sg22F/22A, and st39. Nine strains of three non-pneumococcal species (*S. oralis, S. mitis*, and *S. parasanguinis*) (7 from A-OP, 1 from both A-NP and A-OP, and 1 from C-NP) were each cmPCR-positive for one of 7 serotypes/serogroups (st5, st13, sg15A/15F, sg10F/10C/33C, sg33F/33A/37, sg18C/18A/18B/18F, sg12F/12A/12B/ 44/46) with amplicons revealing 83.6–99.7% sequence identity to pneumococcal references. In total, 150 cmPCR amplicons from carriage specimens were sequenced, including 25 from *lyt*A-negative specimens. Amplicon sequences derived from specimens yielding a pneumococcal isolate with the corresponding serotype were identical or highly conserved (>98.7%) with the reference cmPCR amplicon for the st, while cmPCR amplicons from *lytA*-negative specimens were generally more divergent. Separate testing of 56 A-OPs and 56 A-NPs revealed that ∼94% of the positive cmPCR results from A-NP/OPs were from OP microbiota. In contrast, A-NPs yielded >2-fold more pneumococcal isolates than A-OPs. Verified and suspected non-pneumococcal cmPCR serotypes/serogroups appeared to be relatively rare in C-NPs and A-NPs compared to A-OPs. Our findings indicate that non-pneumococcal species can confound serotype-specific PCR and other sequence-based assays due to evolutionarily conserved genes most likely involved in biosynthesis of surface polysaccharide structures.

## Introduction

The primary reservoir for the opportunistic pathogen *Streptococcus pneumoniae* is the upper respiratory tract, where it coexists in varying proportions with other microbial species. Deep sequencing approaches have revealed that bacterial microbiota complexity varies markedly between the oropharygeal and nasopharyngeal niches, with the more densely colonized oropharynx revealing more bacterial diversity (Charlson et al., 2010; Biesbroek et al., 2012). According to one study of adults, more than 50 different bacterial genera significantly varied in abundance between nasopharyngeal and oropharyngeal sites (Charlson et al., 2010). Pneumococcal disease generally occurs subsequent to carriage in the upper respiratory tract (Gray, Converse & Dillon, 1980). Consistent with data indicating that children constitute a major pneumococcal reservoir (Hendley et al., 1975) is the fact that vaccinating young children with conjugate vaccines significantly decreases disease caused by vaccine serotypes in adults (Whitney et al., 2003). Studies of pneumococcal carriage serotype distributions, especially in young children, have revealed a great deal regarding the potential usefulness and impact of current multivalent conjugate vaccines that target small subsets of the >92 known pneumococcal capsular serotypes (Weinberger, Malley & Lipsitch, 2011), however, the complex biology of pneumococcal carriage is still poorly understood, especially as it pertains to culture-based detection rates that differ markedly between different disease-causing serotypes. Most available respiratory tract pneumococcal serotype distribution data have been obtained from studies of young children. Moreover, few studies of carriage have employed pneumococcal isolation independent, PCR-based detection of pneumococcal serotypes, especially in adults.

Recently, we observed that culture in enriched broth media before plating enhanced both isolation-independent conventional multiplexed PCR (cmPCR)-based and pneumococcal isolation-based detection of pneumococcal nasopharyngeal (NP) carriage serotypes from young children (Carvalho et al., 2010). More recently we applied this methodology to combined NP/oropharyngeal (OP) specimens from adults (A-NP/OPs) and NP specimens from children (C-NPs) living in an area of high HIV prevalence (Carvalho et al., 2012). While the isolation-based and cmPCR data from C-NPs closely approximated results from our previous study (Carvalho et al., 2010), we found an unexpected number and range of serotype detections from cmPCR testing of A-NP/OPs in this population. Based on a combination of putative pneumococcal serotype-specific amplicon and real-time PCR pneumococcal detection data, we hypothesized that non-pneumococcal strains carrying homologs of pneumococcal serotype-specific loci were yielding false cmPCR-determined serotypes (Carvalho et al., 2012). Recent access to the original, stored NP and OP specimens has allowed us to demonstrate here that the abundance and diversity of A-NP/OP –derived cmPCR amplicons within this particular area is primarily due to the presence of diverse strains of oropharyngeal non-pneumococcal mitis group species.

## Materials and Methods

### Specimen collection

This study was approved by both KEMRI and CDC ethical committees. Written informed consent in the local dialect was obtained for specimen collection. Specimens were collected as part of a study to provide baseline data for assessing the direct and indirect impact of introduction of pneumococcal conjugate vaccine on carriage of pneumococci. After obtaining informed consent, nasopharyngeal specimens (NPs) were collected using calcium alginate swab (Fisher Scientific, Pittsburg, PA) at Lwak Mission Hospital in Rarieda District in western Kenya or Tabitha Clinic in Kibera within Nairobi, Kenya during October–December 2009 from 237 healthy children less than 5 years of age (C-NPs). NP swab and oropharyngeal swab specimens (OPs) were collected from 158 consenting adults (A-NPs and A-OPs) at Lwak; of these, 118 (75%) were tested as human immunodeficiency virus (HIV) positive and 40 tested negative for HIV (The design of the carriage survey purposefully oversampled HIV-positive adults). For permanent storage, the individual NP and OP swabs were placed in separate storage/transport vials containing 1.0 ml milk-tryptone-glucose-glycerol (STGG) medium (O’Brien et al., 2001), maintained on wet ice for up to 4 h, and frozen at −70C. Before freezing, NP-STGG and OP-STGG specimens were vortexed for 10 s to disperse organisms from the swab. All NP-STGG and OP-STGG specimens were sent on dry ice to the Kenya Medical Research Institute (KEMRI-CDC) in Kisumu for pneumococcal isolation and for storage.

### Phase 1. Pneumococcal Isolation, serotyping, conventional multiplexed PCR-serotyping (cmPCR), and real time *lytA* PCR

Pneumococcal isolation from all NP-STGG and OP-STGG specimens was performed at KEMRI as previously described (Carvalho et al., 2010). Briefly, supplemented Todd-Hewitt broth (STHB) consisted of 5 ml of Todd-Hewitt broth containing 0.5% yeast extract combined with 1 ml of rabbit serum. After a brief complete thawing and vigorous 10 s vortexing of the NP-STGG and OP-STGG specimens, 200-µl aliquots from children NP-STGG specimens were added to 6 ml STHB. These specimens from children will be referred to as C-NPs. For adults, NP-STGG (200 µl) and OP-STGG (200 µl) aliquots from the same individual were inoculated simultaneously into 6 ml STHB; these combined specimens from adults will be referred to as A-NP/OPs. The C-NPs and A-NP/OPs were incubated for 6 h at 37°C in a CO_2_ incubator prior to streaking onto blood agar plates. One milliliter aliquots of the incubated A-NP/OPs and C-NPs were frozen for subsequent DNA extraction, cmPCR for 40 serotypes or serogroups, and real time *lytA* PCR at Atlanta-CDC as previously described (Carvalho et al., 2010, see http://www.cdc.gov/ncidod/biotech/strep/pcr.htm for latest updates).

Immediately after the 6 h incubation, 10 µl from the A-NP/OPs and C-NPs were streaked onto a blood agar plate at KEMRI-CDC. After incubation at 37°C in a CO_2_ incubator for 18–24 h, alpha-hemolytic colonies (one picked from each colony morphology (Carvalho et al., 2010)) were subcultured and subsequently tested for optochin susceptibility and bile solubility under CO_2_ atmosphere as described (Arbique et al., 2004). Only one colony that represented each colony morphology was picked for pneumococcal identification and serotyping, since we have found no improvement in detecting mixed pneumococcal carriage through picking colonies that share identical appearance (Carvalho et al., 2010). Pneumococcal–positive (optochin-sensitive or bile soluble) isolates recovered at KEMRI-CDC were sent to Atlanta-CDC for serotyping with CDC antisera, which is used to resolve 92 different serotypes, including serotypes 6C and 6D (Melnick, Thompson & Beall, 2010; Mercado et al., 2011).

### Phase 2

Subsequent to the work described above, the original STGG-NPs and STGG-OPs from children and adults were shipped to Atlanta-CDC for isolation/characterization of cmPCR-positive non-pneumococcal species, and for comparison of pneumococcal isolation and cmPCR analysis from a subset of the 158 matched adult specimens (56 STGG-NPs and 56 STGG-OPs) after performing broth-enrichment in STHB as described above in phase 1. Isolated non-pneumococcal strains were also subjected to cmPCR and the real time *lytA* PCR assays as previously described (Carvalho et al., 2010; Carvalho et al., 2007).

### cmPCR amplicon sequencing

cmPCR amplicons from either bacterial isolates or preculture broth extracts were sequenced employing cmPCR primers and the Big Dye V1.1 dideoxy sequencing kit (ABI) on an ABI-3100 sequencer. Reference amplicon sequence coordinates from GenBank accessions are provided at http://www.cdc.gov/ncidod/biotech/files/pcr-oligonucleotide-primers.pdf. The 47 amplicon sequences from this study that do not exactly match the relevant reference sequences are provided in Table S1. cmPCR sequence subtypes encountered in this study that were not previously documented in the public GenBank were designated as subject number targeted serotype or serogroup. For example, cmPCR sequence subtype 300.2 corresponds to adult subject 300 and a positive cmPCR amplicon for serotype 2 exactly matching in length with the reference cmPCR amplicon referred to in the CDC cmPCR primer list (http://www.cdc.gov/ncidod/biotech/files/pcr-oligonucleotide-primers.pdf, primer sequences, GenBank accessions and base coordinates are included in this list). Similarly, 300.10 refers to a cmPCR amplicon sequence corresponding to serogroup 10F/10C/33C. Designations that share complete sequence identity to the indicated GenBank accession over the described base coordinates of the pneumococcal reference sequence are preceded by “st” and the pneumococcal serotype without any reference to the specimen number. For example, st14.14, st10F.10, st3.3 all represent sequence identity within amplicon overlaps with sequences previously documented in GenBank that were derived from pneumococci of the indicated serotypes.

### Species approximation of non-pneumococcal strains

Multilocus amplification/sequencing and nearest species matches were determined at http://viridans.emlsa.net/ for each cmPCR-positive non-pneumococcal isolate by multilocus sequence analysis as described (Bishop et al., 2009). This site automatically concatenates 7 entered housekeeping locus sequences (*map*, 426 bp; *pfl*, 351 bp; *ppaC*, 552 bp; *pyk*, 492 bp; *rpoB*, 516 bp; *sodA*, 378 bp; *tuf*, 426 bp) in order to compare a 3063 bp sequence with the online species database (Bishop et al., 2009). A dendrogram of the 9 concatenated 3063 bp sequences, together with corresponding concatenates from two pneumococcal strains, was constructed using the Wisconsin Package (Wisconsin Package, version 10.3 Accelrys Inc., San Diego, CA) Distances program with the neighbor-joining approach and the un-corrected distance model.

### Specimen information

Specific information pertaining to individual specimens (of the 237 C-NPs and 158 A-NP/OPs described) is available upon request. This also includes the 56 NP-STGG and 56 OP-STGG adult specimens that were cross-compared to original combined co-cultured results. Sequence subtypes of cmPCR amplicons shorter than 200 bp are included within Table S1 (due to current GenBank policy barring their inclusion). GenBank accessions KC771356–KC771416 represent all additional cmPCR amplicon sequences >200 bp in length from this study except for those noted as being already documented within GenBank. All housekeeping locus sequences were deposited in GenBank with accessions KC808157–KC808165 and KC779228–KC779254.

## Results

### Culture based pneumococcal carriage and cmPCR serotyping results

In children, we found an 85.2% (202/237) NP carriage frequency, with isolation of pneumococci of 40 different sts and 9 isolates that were non-serotypeable (Tables 1 and 2). In 118 HIV-positive adults, pneumococcal isolation results revealed an NP/OP carriage frequency of 40.7% with 20 sts (Tables 1 and 3). Among A-NP/OPs from 40 HIV-negative individuals, 5 were culture-positive for one of 5 sts (4, 11A, 18C, 19F, 23F) for a carriage frequency of 12.5% (Tables 1 and 4). Sts 19F and 23F were the most frequently culture-derived sts in C-NPs, while 19F and 11A were the most common among HIV-positive adult NP/OP specimens (A-NP/OPs) (Tables 1 and 3). Nineteen of the 20 serotypes recovered from A-NP/OPs were also found in children, with the exception of 7A, recovered from a single HIV-positive A-NP/OP.

**Table 1:** Summary of pneumococcal culture, *lytA* testing, and cmPCR-testing of carriage specimens from children and adults.

	Children NPs (*n* = 237)	HIV + adult NP/OPs (*n* = 118)	HIV− adult NP/OPs (*n* = 40)
No. pneumococcal culture + (%)	202 (85.2)	48 (40.7)	5 (12.5)
No. *lytA* + (%)	221 (93.2)	105 (89.0)	24 (60.0)
No. cmPCR + (%)	219 (92.4)	116 (98.3)	38 (95.0)
No. *lytA*− and cmPCR− (%)	14 (5.9)	1 (0.8)	2 (5.0)
cmPCR + , *lytA*− (%)	2 (0.8)	12 (10.2)	14 (35.0)
pneumococcal culture + , *lytA*− (%)	0	0	0

**Table 2:** Pneumococcal isolation-based serotyping results in children (*n* = 237) with corresponding cmPCR results.

Serotypes detected by culture (corresponding sts co-detected by cmPCR)	No. Isolation/Quellung-positive (percentage of specimens)	No. cmPCR-positive (percentage of specimens)/ number corresponding to positive Quellung (percentage of Quellung-positive specimens)	Ratio of cmPCR-positives/ culture Quellung-positive
19F (19F)	30^a^ (12.2)	37 (15.7)/30 (100)	1.2
23F (23F)	19 (8.0)	31 (13.1)/17 (89.5)	1.6
6A (6A/6B)	15^a^ (6.4)	42 (17.7)/27 (96.4)	1.5
6B (6A/6B)	13 (5.5)		
14 (14)	11 (4.6)	28 (11.8)/11 (100)	2.5
11A (11A/11D)	9^a^ (3.8)	13 (5.5)/9 (100)^a^	1.4
19B (not in cmPCR assay)	7 (3.0)	0	0
1 (1)	6^a^ (2.5)	9 (3.8)/6 (100)	1.5
13 (13)	6 (2.5)	8 (3.4)/4 (66.7)	1.3
20 (20)	5 (2.1)	7 (3.0)/5 (100)^a^	1.4
15B (15B/15C)	5 (2.1)	12 (5.1)/10 (100)^b^	1.2
15C (15B/15C)	5^a^ (2.1)		
34 (34)	5^a^ (2.1)	9 (3.8)/5 (100)	1.8
10A (10A)	4 (1.7)	6 (2.5)/4 (100)^b^	1.5
23B (23B)	4 (1.7)	7 (3.0)/4 (100)	1.8
35B (35B)	4 (1.7)	8 (3.4)/4 (100)	2.0
3 (3)	3^a^ (1.3)	6 (2.5)/2 (66.7)	2.0
18A (18A/18B/18C/18D)	3 (1.3)	8 (3.4)/4 (100)	1.8
18C (18A/18B/18C/18D)	1 (0.4)		
17F (17F)	3 (1.3)	4 (1.7)/3 (100)^b^	1.3
23A (23A)	3^a^ (1.3)	3 (1.3)/3 (100)^a^	1.0
16F (16F)	3 (1.3)	8 (3.4)/3 (100)	2.7
15A (15A/15F)	3 (1.3)	11 (4.6)/3 (100)^a^	3.7
4 (4)	3 (1.3)	8 (3.4)/3 (100)	2.7
5 (5)	2 (0.8)	2 (0.8)/ 2 (100)	1.0
7F (7F/7A)	2 (0.8)	5 (2.1)/2 (100)	2.5
9V (9V/9A)	2 (0.8)	2 (0.8)/2 (100)	1.0
12F (12F/12A/44/46)	2 (0.8)	13 (5.5)/4 (100)	3.2
46 (12F/12A/44/46)	2 (0.8)		
38 (38/25F/25A)	2 (0.8)	9 (3.8)/2 (66.7)^a^	3.0
25A (38/25F/25A)	1 (0.4)		
21 (21)	2^a^ (0.8)	6 (2.5)/ 2 (100)	3.0
6C (6C/6D)	1 (0.4)	1 (0.4)/1 (100)	1.0
9L (9N/9L)	1 (0.4)	1 (0.4)/1 (100)	1.0
10F (10F/10C/33C)	1 (0.4)	13 (5.5)/1 (100)	13.0
19A (19A)	1 (0.4)	8 (3.4)/1 (100)	8.0
24B (24A/24B/24F)	1 (0.4)	7 (3.0)/1 (100)	7.0
28F (not in mPCR assay)	1 (0.4)	0	0
35A (35A/35C/42)	1 (0.4)	2 (0.8)/1 (100)	2.0
35F (35F/47F)	1 (0.4)	5 (2.1)/1 (100)	5.0
(2)	0	0/1 (0)	0
nontypeable (PCR-NT)	9^a^ (3.8)	16^c^ (6.8)	1.8

**Notes.**

a Found in combination with 1 other serotype in 1–3 specimens.

b 1–3 culture-positive specimen(s) initially found cmPCR-negative for the corresponding serotype were retested using monoplex PCR reaction and were subsequently found positive.

c Specimens positive for *cpsA* positive control and negative for serotype or serogroup.

**Table 3:** Isolation and Quellung reaction based serotyping results in HIV-positive adults (*n* = 118) with corresponding cmPCR results.

Serotypes detected by culture (corresponding sts co-detected by cmPCR)	No. Isolation/Quellung-positive (percentage of specimens)	No. cmPCR-positive (percentage of specimens)/ number corresponding to positive Quellung (percentage of Quellung-positive specimens)	Ratio of cmPCR-positives/ Qculture Quellung-positive
19F (19F)	7 (5.9)	13 (11.0)/7 (100)	1.9
11A (11A/11D)	5 (4.2)	11 (9.3)/5 (100)	2.2
3 (3)	5 (4.2)	9 (7.6)/4 (80)	1.8
6A (6A/6B)	4 (3.4)	7 (5.9)/5 (100)	1.4
6B (6A/6B)	1 (0.8)		
16F (16F)	4 (3.4)	5 (4.2)/4 (100)	1.2
13 (13)	3 (2.5)	9 (7.6)/3 (100)	3.0
4 (4)	2 (1.7)	6 (5.1)/2 (100)	3.0
23F (23F)	2 (1.7)	5 (4.2)/2 (100)	2.5
21 (21)	2 (1.7)	17 (14.4)/1 (50)	8.5
15B (15B/15C)	2 (1.7)	5 (4.2)/2 (100)	2.5
1 (1)	2 (1.7)	3 (2.5)/2 (100)	1.5
34 (34)	2 (1.7)	6 (5.1)/2 (100)	3.0
18C (18C/18A/18B/18F)	1 (0.8)	70 (59.3)/2 (100)	35.0
18A (18C/18A/18B/18F)	1 (0.8)		
7F (7F/7A)	1 (0.8)	7 (5.9)/2 (100)	3.5
7A (7F/7A)	1 (0.8)		
14 (14)	1 (0.8)	4 (3.4)/1 (100)	4.0
35B (35B)	1 (0.8)	6 (5.1)/1 (100)	6.0
15A (15A/15F)	1 (0.8)	6 (5.1)/1 (100)	6.0
(10F/10C/33C)^a^	0	92 (78.0)	
(33F/33A/37)^a^	0	57 (48.3)	
(2)	0	38 (32.2)	
(39)	0	38 (32.2)	
(20)	0	37 (31.3)	
(5)	0	35 (29.7)	
(35A/35C/42)^a^	0	22 (18.6)	
(10A)	0	21 (17.8)	
(22F/22A)^a^	0	20 (16.9)	
(17F)	0	20 (16.9)	
(9N/9L)^a^	0	13 (11.0)	
(24A/24B/24F)^a^	0	11 (9.3)	
(12F/12A)^a^	0	9 (7.6)	
(7C/7B/40)^a^	0	5 (4.2)	
(8)	0	4 (3.4)	
(31)	0	1 (0.8)	
Nontypeable (PCR-NT)	0	3 (2.5)^b^	

**Notes.**

a All individual component serotypes are identifiable by Quellung reaction.

b PCR-NT defined as detection of only the *cpsA* control amplicon without detection of any serotype/serogroup-specific amplicons.

**Table 4:** Isolation and Quellung reaction based serotyping results in HIV-negative adults (*n* = 40) with corresponding cmPCR results.

Serotypes detected by culture (corresponding sts co-detected by cmPCR)	No. Isolation/Quellung-positive (percentage of specimens)	No. cmPCR-positive (percentage of specimens)/number corresponding to positive Quellung (percentage of Quellung-positive specimens)	Ratio of cmPCR-positives/ culture Quellung-positive
4 (4)	1 (2.5)	4 (10.0)/1 (100)	4.0
11A (11A/11D)	1 (2.5)	3 (7.5)/1 (100)	3.0
18C (18C/18A/18B/18F)	1 (2.5)	24 (60.0)/1 (100)	24.0
19F (19F)	1 (2.5)	1 (2.5)/1 (100)	1.0
23F (23F)	1 (2.5)	2 (5.0)/1 (100)	2.0
(10F/10C/33C)^a^	0	35 (87.5)	
(33F/33A/37)^a^	0	30 (75.0)	
(20)	0	23 (57.5)	
(5)	0	16 (40.0)	
(10A)	0	15 (37.5)	
(39)	0	15 (37.5)	
(7F/7A)^a^	0	13 (32.5)	
(2)	0	12 (30.0)	
(22F/22A)^a^	0	12 (30.0)	
(9N/9L)^a^	0	12 (30.0)	
(35A/35C/42)^a^	0	10 (25.0)	
17F	0	10 (25.0)	
21	0	10 (25.0)	
(12F/12A)^a^	0	8 (20.0)	
(13)	0	5 (12.5)	
(35B)	0	4 (10.0)	
(34)	0	3 (7.5)	
(24A/24B/24F)^a^	0	3 (7.5)	
(6A/6B)^a^	0	1 (2.5)	
(3)	0	1 (2.5)	
(15A/15F)^a^	0	1 (2.5)	
(9V/9A)^a^	0	1 (2.5)	

**Notes.**

a All individual component serotypes are identifiable by Quellung reaction.

In C-NPs the ratios of total cmPCR-positive/Quellung results ranged from 1.0 to 13.0 (Table 2, column 4). The three highest ratios (13.0, 8.0, and 7.0) were restricted to Quellung serotypes found only from single isolates (10F, 19A, and 24B). For serotypes representing multiple pneumococcal isolates this ratio ranged from 1.0–3.7. The cmPCR serotype/Quellung serotype ratios were generally greater from A-NP/OPs than from C-NPs (Tables 2–4). The greatest ratio was shown for sts 18A and 18C, identified by Quellung in 2 HIV-positive adults, which corresponded to cmPCR-positive A-NP/OPs for sg18 from 70 individuals in this population (Table 3). Among A-NP/OPs there were 39 instances involving 21 different cmPCR types where we found a lack of any corresponding pneumococcal isolation (Quellung) -based results (Tables 3 and 4); among children, there was only 1 such instance. This trend was apparent in both HIV-positive and HIV-negative adults, with the 2 most prevalent examples in both groups being cmPCR types 10F/10C/33C (78.0–87.5% frequency) and 33F/33A/37 (48.3%–75% frequency) (Tables 3 and 4).

### Real time PCR (*lytA*) and cmPCR results

Overall, more carriage specimens were positive for the presence of pneumococci using real-time *lytA* PCR than by culture. While among children the number of *lytA*-positive C-NPs was close to the number positive by culture (93.2% compared to 85.2%, respectively) (Table 1), results differed when comparing the 2 parameters from the A-NP/OPs (89.0% *lytA*-positive vs 40.7% culture-positive in HIV + and 60% *lytA*-positive vs.12.5% culture-positive in HIV −).

Testing A-NP/OPs with cmPCR produced many more positive results compared to culture. While, again, there was a modest increase in the number of cmPCR-positive C-NPs compared to culture positive C-NPs (92.4% vs 85.2%), there was approximately 2.4-fold more cmPCR-positive A-NP/OPs in the HIV-positive set (98.3% vs 40.7%) and 7.6-fold more cmPCR-positive A-NP/OPs in the HIV-negative set (95.0% vs 12.5%).

We found close agreement in the number of positive C-NPs when tested by cmPCR and by *lytA*, however, in A-NP/OPs there was a notable increase of cmPCR-positive specimens compared to *lytA*-positive specimens. Another observation that markedly differed between C-NPs and A-NP/OPs was the relatively large numbers of cmPCR-positive A-NP/OPs samples that were also *lytA*-negative (two C-NPs (0.8%) vs 12 (10.2%) of HIV + A-NP/OPs vs 14 (35.0%) of HIV-negative A-NP/OPs; Table 1).There were very large numbers of co-carried cmPCR types in A-NP/OPs (up to 11–12 with average of 5.3–6.5) compared to C-NPs (maximum of 5 with average of 1.5) (Table 5).

**Table 5:** Distribution of numbers of carried cmPCR types and association with *lytA*-negative specimens.

Study group	Number of cmPCR types (% of total)/No. *lytA*-negative														Avg. No. of carried cmPCR types^b^
	0	PCR-NT^a^	1	2	3	4	5	6	7	8	9	10	11	12	
HIV positive Adults (NP/OP) *n* = 118	2 (1.7)/1	3 (2.5)/0	11 (9.3)/0	12 (10.2)/2	5 (4.2)/0	14 (11.9)/3	16 (13.6)/4	16 (13.6)/1	17 (14.4)/1	7 (5.9)/0	5 (4.2)/0	4 (3.4)/1	1 (0.8)/0	5 (4.2)/0	5.3
HIV negative Adults (NP/OP) *n* = 40	2 (5.0)/1	0	0	0	1 (2.5)/1	6 (15.0)/2	4 (10.0)/3	4 (10.0)/3	10 (25.0)/4	3 (7.5)/1	5 (12.5)/0	4 (10.0)/0	1 (2.5)/0	0	6.5
Healthy Children (NP) *n* = 237	16 (6.8)/14	16 (6.8)/0	98 (41.4)/1	72 (30.4)/1	27 (11.4)/0	6 (2.5)/0	2 (0.8)/0	0	0	0	0	0	0	0	1.5

**Notes.**

a PCR-NT defined as detection of only the *cpsA* control amplicon without detection of any serotype/serogroup-specific amplicons.

b Total number of subjects divided by total number of the 1–12 carried cmPCR types.

Identifying cmPCR types within *lytA*-negative specimens was an unexpected finding (Table 5). To verify these findings, we sequenced 28 amplicons from 13 different *lytA*-negative A-NP/OPs representing 13 different cmPCR types and 26 sequence subtypes (Table 6). Each of the 28 amplicons shared the exact same length as the pneumococcal reference amplicon for the serotype (38–628 bp), and each subtype displayed 86–100% sequence identity to the targeted pneumococcal serotype.We verified the most extreme example of cmPCR type diversity within a single *lytA*-negative A-NP/OP; this specimen (No. 300) contained 10 different cmPCR types. We obtained all 10 amplicon sequences corresponding to all 10 observed cmPCR types, including subtypes 300.2, 300.5, 254.7, 300.10A, 300.10, 269.18, st39.39, 300.17F, 300.9, and 329.33. These subtypes exhibited a range of 91.4–100% sequence identity to published pneumococcal reference amplicons of corresponding serotypes (Table 6).

**Table 6:** Associations of specific cmPCR amplicon sequence subtypes with pneumococci, non-pneumococcal species, or unknown species based upon culture and *lytA* assay findings.

cmPCR type (total combined positive A-NP/OPs and C-NPs from study)^a^	Amplicon length in bp	cmPCR amplicon sequence subtype^b^ (No. sequenced)	%amplicon sequence identity with Spn reference	No. specimens characterized by^c^						Culture and/or *lytA*-based species association^d^ (Spn serotype)
				Cultured Spn of the corresponding serotype	Cultured non-Spn carrying sequence	*lytA*+	*lytA*-	Adult NP/OP	Child NP	
1 (14)	214	st1.1 (3)	100	1	0	3	0	2	1	*S. pneumoniae* (1)
2 (56)	230	378.2 (2)	97.8	0	0	1	1	2	0	non-Spn
		291.2 (1)	97.4	0	0	1	0	1	0	unknown
		300.2 (1)	97.0	0	0	0	1	1	0	non-Spn
		327.2 (1)	97.8	0	0	1	0	1	0	unknown
3 (16)	309	41.3 (7)	98.7	2	0	7	0	3	4	Spn (3)
		st3.3 (1)	100	0	0	1	0	1	0	unknown
4 (18)	368	st4.4 (5)	100	1	0	5	0	3	2	Spn (4)
		394.4 (3)	94.6	0	0	2	1	3	0	non-Spn
5 (53)	295	361.5 (6)	98.6	0	0	5	1	6	0	non-Spn
		st5.5 (3)	100	2	0	3	0	0	3	Spn (5)
		294.5 (1)	96.6	0	1	1	0	1	0	*S. mitis*
		90.5 (1)	96.9	0	0	1	0	0	1	unknown
		283.5 (1)	96.3	0	0	1	0	1	0	unknown
		300.5 (1)	99.0	0	0	0	1	1	0	non-Spn
		392.5 (1)	99.3	0	0	1	0	1	0	unknown
6AB (50)	191	141.6 (6)	97.9	0	0	6	0	0	6	unknown
		st6A.6 (7)	100	1	0	7	0	1	6	Spn (6A)
		359.6 (2)	99.0	1	0	2	0	2	0	Spn (6B)
		267.6 (1)	99.5	0	0	1	0	1	0	unknown
7FA (25)	546	254.7 (2)	97.1	0	0	1	1	2	0	non-Spn
		274.7 (1)	97.8	0	0	0	1	1	0	non-Spn
		377.7 (1)	98.2	0	0	1	0	1	0	unknown
		398.7 (1)	98.4	0	0	0	1	1	0	non-Spn
		st7F.7 (1)	100	1	0	1	0	1	0	Spn (7F)
9NL (26)	464	119.9 (1)	99.4	1	0	1	0	0	1	Spn (9L)
		300.9 (1)	97.8	0	0	0	1	1	0	non-Spn
9VA (3)	759	st9V.9 (1)	100	1	0	1	0	0	1	Spn (9V)
10A (42)	567	47.10A (1)	99.8	1	0	1	0	0	1	Spn (10A)
		300.10A (1)	96.1	0	0	0	1	1	0	non-Spn
10F/10C/33C (140)	192	49.10 (2)	92.7	0	0	1	1	1	1	non-Spn
		32.10 (1)	92.7	0	2^d^	0	1	0	1	non-Spn
		300.10 (1)	92.7	0	0	0	1	1	0	non-Spn
		st10F.10 (1)	100	1	0	1	0	0	1	Spn (10F)
		248.10 (1)	93.2	0	0	1	0	1	0	unknown
		265.10 (1)	92.7	0	0	1	0	1	0	unknown
		378.10 (2)	91.7	0	2	1	2	2	0	*S. oralis*
		387.10(1)	93.8	0	1	0	1	1	0	*S. parasanguinis*
12FAB/44/46 (30)	321	376.12(1)	99.7	0	1	0	1	1	0	*S. oralis*
13 (22)	596	375.13 (1)	83.6	0	1	0	1	1	0	*S. oralis*
		321.13 (1)	99.5	1	0	1	0	1	0	Spn (13)
14 (32)	130	st14.14 (11)	100	1	0	11	0	3	8	Spn (14)
15AF (12)	362	276.15 (1)	87.8	0	1	0	1	1	0	*S. oralis*
17F (34)	628	st17F.17F(1)	100	1	0	1	0	0	1	Spn (17F)
		300.17F (1)	91.4	0	0	0	1	1	0	non-Spn
18CABF (102)	511	257.18 (2)	90.4	0	1	1	1	1	1	*S. mitis*
		269.18 (4)	92.5	0	0	2	2	4	0	non-Spn
		368 (1)	91.1	0	0	1	0	1	0	unknown
		st18A.18 (6)	100	4	0	6	0	1	5	Spn (18A)
		st18C.18 (4)	100	3	0	4	0	2	2	Spn (18C)
19A (8)	511	st19A.19A(3)	100	1	0	3	0	0	3	Spn (19A)
19F (51)	241	st19F.19F(9)	100	1	0	9	0	3	6	Spn (19F)
22F/22A (32)	579	276.22 (3)	95.7	0	0	2	1	3	0	non-Spn
		395.22 (2)	94.3	0	0	2	0	2	0	unknown
		344.22 (1)	95.9	0	0	0	1	1	0	non-Spn
23F (38)	319	st23F.23F(11)	100	1	0	11	0	2	9	Spn (23F)
33F/33A/37 (87)	280	329.33 (3)	96.4	0	0	2	1	3	0	non-Spn
		383.33 (2)	98.2	0	0	2	0	2	0	unknown
		291.33 (1)	96.8	0	1	1	0	1	0	*S. oralis*
		344.33 (1)	97.9	0	0	0	1	1	0	non-Spn
39 (53)	38	st39.39 (1)	100	0	0	0	1	1	0	non-Spn

**Notes.**

a Derived from adding positive cmPCR specimens from the 395 total specimens listed in column 3 of Tables 2–4.

b Subtype designations start with “st” for sequences that share complete identity to the corresponding pneumococcal reference (for listing of GenBank accessions and coordinates, see http://www.cdc.gov/ncidod/biotech/files/pcr-oligonucleotide-primers.pdf).

c Gray shaded boxes indicate data associating cmPCR sequence subtypes with pneumococcal or non-pneumococcal sources.

d Although not found in these study isolates, was previously observed within 2 independent laboratory reference strains of *S. oralis* (Bishop et al., 2009).

**Figure 1:**
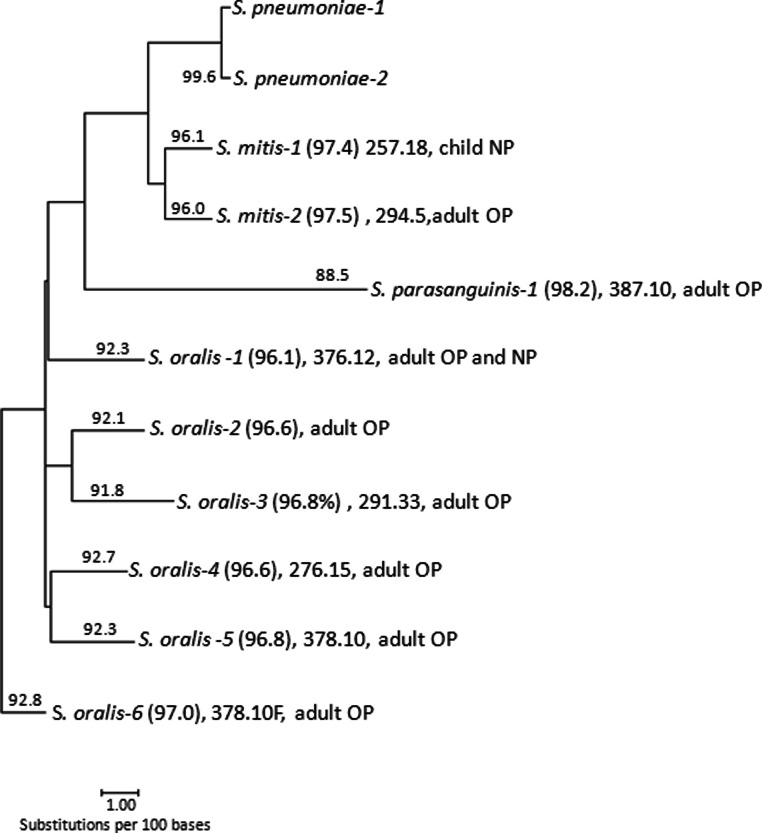
Phylogenetic analysis of 3063 bp concatenated housekeeping gene fragments from 9 non-pneumococcal. cmPCR+ strains isolated during this study (indicated as S. mitis-1, etc.). Numbers to left indicate % sequence identity of concatenated 3063 bp from strain compared to represented S. pneumoniae strain at top of dendrogram. Numbers to right of strain (parenthesis) indicates percent identity of the 3063 bp sequence to the closest matching MLSA database strain (http://viridans.emlsa.net/). Also included are strain cmPCR sequence subtype designations (from Table 6) and specimen source. Dendrogram was created by subjecting aligned 3063 sequences to the neighbor-joining approach using the uncorrected distance method.

Two putatively non-pneumococcal cmPCR amplicon sequence subtypes, 32.10 and 257.18, were identified from the only two *lytA*-negative, cmPCR-positive C-NP specimens. Although causal subtype 32.10 bacterial strains were not recovered from specimens in this study, this subtype was previously found within two different lab reference strains of *S. oralis* (Carvalho et al., 2012). Sequence subtype 257.18 was recovered from both an A-NP/OP and a C-NP, from which the causal non-pneumococcal strain was isolated and identified as *S. mitis* on the basis of multilocus sequence analysis (Table 6, Fig. 1).

### cmPCR amplicon features

We found all 40 cmPCR assay types (Carvalho et al., 2010) within these 395 study specimens (Tables 2–4). Table 6 depicts sequence data from 153 amplicons that represent 23 cmPCR types chosen for sequence analysis. Within these 23 cmPCR types, a total of 61 sequence subtypes were found. For 19 of the 61 subtypes, each representing 1–11 specimens, matching Quellung-based and cmPCR-based findings were obtained (for sts 1, 3, 4, 5, 6A, 6B, 7F, 9L, 9V, 10A, 10F, 13, 14, 17F, 18A, 18C, 19A, 19F, and 23F). Fifteen of these 19 cmPCR subtypes (st1.1, st4.4, st5.5, st6A.6, st7F.6, st9V.9, st10F.10, st13.13, st14.14, st17F.17F, st18A.18, st18C.18, st19A.19A, st19F.19F, and st23F.23F) shared sequence identity over their 130–759 bp overlap with the corresponding pneumococcal reference amplicons. For serogroup 18, the slight sequence differences between the 18A and 18C amplicons (share 97.6% identity over 511 bp) were in each of 7 instances predictive of serotype 18A (4 instances) or of serotype 18C (3 instances). Four sequence subtypes– 41.3, 359.6, 119.9, and 47.10A– displayed minor sequence differences compared to the reference subtypes even though they were found within pneumococcal isolates of corresponding Quellung-derived serotypes (Table 6) and also were found within the corresponding specimen DNA extracts in an isolation-independent manner. Only one subtype with sequence identity to its respective pneumococcal serotype reference sequence (st39.39) was associated with a non-pneumococcal source, however, this cmPCR amplicon was only 38 bp after subtraction of PCR primer sequences (Table 6). We were unable to associate the cmPCR subtype st3.3 with a corresponding pneumococcal isolate, although it shared sequence identity to the pneumococcal reference sequence for the serotype.

Twenty-six cmPCR subtypes, corresponding to 15 cmPCR types, were associated with *lytA*-negative specimens, suggestive of non-pneumococcal species (Table 6). As shown in Fig. 1, each of the 8 subtypes associated with one of 9 distinct non-pneumococcal strains represented a distinct strain within one of 3 different mitis group species, with the exception of 378.10 which was found in two genetically distinct *S. oralis* strains. Within this limited sampling of individual cmPCR types, an unexpected degree of sequence diversity was observed. While all 10 cmPCR-18C/A/B/F positive specimens that corresponded to st18A or st18C pneumococci displayed sequence identity to st18A and st18C reference amplicons, respectively, the remaining 7 cmPCR-18C/A/B/F positive specimens represented 3 quite divergent sequence subtypes described earlier (Carvalho et al., 2012), including 257.18 that was 90.5% identical to the reference sequence. Even more subtype diversity was observed within cmPCR type 10F/10C/33C. The one amplicon sequence examined from a specimen that yielded a serotype 10F pneumococcal isolate was identical to the pneumococcal 10F amplicon reference (Table 6). Nine other cmPCR type 10F/10C/33C amplicons, including 7 from *lytA*-negative specimens, yielded 7 additional diverse cmPCR subtypes that displayed only 92–94% identity to the published pneumococcal reference amplicon (Table 6). In contrast, two of the 6 cmPCR type 5 subtypes that were associated with non-pneumococcal sources (one identified from *S. mitis* and one associated with a *lytA*-negative specimen) and displayed amplicon sequences very similar to the st5 reference sequence (96.6–99% identity) (Table 6, Fig. 1).

Seventeen cmPCR types were found in this study from which representative amplicons were not subjected to sequence analysis (cmPCR types 7C/7B/40, 6C/6D, 8, 11A/11D, 15B/15C, 16F, 20, 21, 23A, 23B, 24A/24B/24F, 31, 34, 35A/35C/42, 35B, 35F/47F, and 38/25F/25A). Of these, cmPCR types 20, 21, 24A/24B/24F, 35A/35C/42, and 35B were found within multiple *lytA*-negative specimens, which suggests their presence in non-pneumococcal carriage strains. Also consistent with this notion was the high numbers of positive specimens for these 5 cmPCR types in A-NP/OPs relative to the isolation of pneumococci expressing these serotypes (Tables 3 and 4; note high cmPCR/Quellung ratios and/or absence of Quellung-based data). For 14 cmPCR subtypes depicted in Table 6 (see last column, “unknown” association), representing 9 cmPCR types, associations with either pneumococcal or non-pneumococcal sources could not be determined due to lack of representation in pneumococcal isolates or in *lytA*-negative specimens.

The cmPCR sequence subtypes st1.1, st19A.19A, st19F.19F, and st23F.23F were encountered multiple (3–11) times in both adult and child specimens that were either positive or negative for pneumococcal isolates of the corresponding serotype (Table 6) and were the only representative sequence subtype of their corresponding serotype. In addition, none of the *lytA*-negative specimens in this study were positive for these 5 cmPCR types.

### The majority of cmPCR-type diversity from adults is derived from oropharyngeal microbiota

We compared the numbers of cmPCR types from corresponding separate OP and NP specimens from 39 adults (24 HIV − and 15 HIV +) (Table 7). We found that A-OP specimens accounted for 91.3–95.6% of the cmPCR types found within corresponding A-NP/OP specimens (94/103 within HIV− adults and 94/103 within HIV + adults). In contrast, within an expanded sampling of 56 subject specimen sets (including the 39 sets tested for cmPCR types), we recovered 2.2–3.5 fold more pneumococcal isolates from A-NP specimens than from corresponding A-OP or combined A-NP/OP specimens (Tables 8A and 8B). Four of 28 (14.3%) NPs and one of 28 OPs (3.6%) from HIV-negative individuals were found to be culture-positive that corresponded to A-NP/OPs originally tested as culture-negative (Table 8A). Similarly, in HIV + individuals, 6 of 25 NPs (24%) that corresponded to originally culture-negative combined NP-OP results were pneumococcal isolation-positive.

**Table 7:** Cross-comparison of cmPCR findings from 39 A-NP/OPs specimens with separate NP and OP specimens.

Study group (No. specimens)	Cumulative No. of positive cmPCR serotype results (range within specimens)				
	Combined NP/OP	OP alone	Shared between NP/OP and OP	NP alone	Shared between NP/OP and NP
HIV− adults (*n* = 24)	161 (3–11) average = 6.7	175 (3–12) average = 7.3	154 (2–11) average = 6.4	5 (1–2) average = 0.2	4 (1–2) average = 0.17
HIV + adults (*n* = 15)	103 (4–12) average = 6.9	118 (5–12) average = 7.9	94 (4–11) average = 6.3	15 (0–8)^a^ average = 1.0	11 (0–7) average = 1.4

**Notes.**

a One specimen among the 15 yielded 8 cmPCR serotypes; all others had 0–1 cmPCR serotypes.

### Non-pneumococcal Mitis group streptococci recovered from A-OP, A-NP, and C-NP specimens

From 56 adults, a total of 448 different non-pneumococcal (optochin-resistant and bile-insoluble; 115 from A-NPs and 333 from A-OPs) isolates were recovered that represented a broad variety of colony types. In addition, we recovered 4 colonies from two *lytA*-negative C-NP specimens in the same manner.

We cmPCR typed all colonies representing different morphologies from the 56 A-OPs (total of 333 colonies) and 56 A-NPs (total of 115 colonies), corresponding to 56 original A-NP/OPs. In addition, we cmPCR typed 4 colonies from the only two *lytA*-negative C-NP specimens that were cmPCR-positive. Cumulatively, we found 9 cmPCR-positive non-pneumococcal strains (1 from C-NP, 7 from A-OP, 1 from A-OP and A-NP of same individual). On the basis of multi-locus sequence analysis of concatenated housekeeping gene fragments, these were identified as *S. oralis* (6 strains, cmPCR subtypes 376.12, 375.13, 291.33, 276.15, and 378.10). *S. mitis* (2 strains, cmPCR types 257.18 and 294.5), and *S. parasanguinis* (1 strain, cmPCR subtype 387.10) (Fig. 1). These 3063 bp sequences shared 91.8–96.1% sequence identity to corresponding sequences from representative pneumococcal strains. All 9 strains were found to be *lytA*-negative, were Quellung-nontypeable, and non-encapsulated when visualized with capsule stain. cmPCR subtypes from two of these non-pneumococcal strains were found in multiple study specimens. cmPCR subtype 257.18, recovered from a C-NP, was also observed from a *lytA*-negative HIV + A-NP/OP (Table 8). cmPCR subtype 378.10 was recovered from 2 genetically distinct *S. oralis* strains (Fig. 1) that were isolated from different A-OP specimens (Table 6).

**Table 8:** Pneumococcal isolation-based serotyping data from cross-compared A-NP/OPs, NPs alone, and OPs from 31 HIV-negative (A) and 25 HIV-positive (B) adults.

Specimen	NP	NP/OP	OP
A			
1	23F	23F	23F
2	18C	18C	18C
3	4	4	neg
4	35C	neg	neg
5	35A	neg	neg
6	34	neg	neg
7	23A	neg	neg
8	neg	neg	35B
9–31	neg	neg	neg
Total positive specimens	7	3	3
B			
32	6B	6B	6B
33	23F	11A	23F
34	13	13	13
35	19F	19F	neg
36	7C	neg	7C
37	neg	11A	11A
38	35A	neg	neg
39	34	neg	neg
40	34	neg	neg
41	3	neg	neg
42	13	neg	neg
43	16F	neg	neg
44–56	neg	neg	neg
Total positive specimens	11	5	5

## Discussion

We have shown a small portion of what is an as yet unquantified upper respiratory reservoir of non-pneumococcal mitis group streptococcal strains that carry homologs of a large percentage of the known pneumococcal serotype or serogroup-specific genes that encode enzymes for specific polymerization and export functions (*wzy* and *wzx* genes; Aanensen et al., 2007). These genes serve as targets for the majority of the 40 individual primer sets that we employ. For example, we suspect that the majority of the cmPCR type 2-positive specimens reflects non-pneumococcal strains, given overall positivity in >30% of A-NP/OPs, while no serotype 2 pneumococcal strains were recovered. It is quite likely that many individual cmPCR sequence subtypes even within the same cmPCR type represent distinct non-pneumococcal strains of one or more species (as judged by MLSA (Fig. 1)). Among only 5 cmPCR type 2 amplicons, we found 4 distinct sequence subtypes (Table 6). The remaining 51 cmPCR-positive specimens are predicted to represent numerous additional type 2 subtypes. Strain and even species diversity within such cmPCR types remains to be investigated. For example, with relatively little sampling we have now found 4 different cmPCR-positive mitis group species for the cmPCR type 10F/10C/33C amplicon (*S. oralis*, *S. parasanguinis*, *S. infantis*, and *S. gordonii*) (this study and Carvalho et al., 2012). In a previous study, we reported associating a *Streptococcus salivarius* (salivarius group) reference strain with this cmPCR type (Carvalho et al., 2012). Subsequently, we have found that our records were in error and that this strain (SS1061) is a strain of the mitis group species *Streptococcus gordonii*.

While culture-independent cmPCR-serotyping of A-OP or combined A-OP/NP specimens added a very large number of false-positive results into our study, this technique also added valuable pneumococcal serotype detection data to the C-NP portion of this study. We believe that culture-independent cmPCR of these enriched specimens added valuable missed data for each of the vaccine-targeted serotypes 19F, 23F, 6A, 6B, 14, 1, 3, 4, and 7F (Table 2). Even though the evidence is not quantitatively supported by pneumococcal isolation data, it does suggest that few, if any, confounding non-pneumococcal amplicon results were obtained for these serotypes. Within C-NPs none of these “cmPCR serotypes” were observed that were not represented in the overall sampling by cultured pneumococci. For each of these targets, amplicon sequences were identical whether they were derived from specimens that were culture-positive for the corresponding pneumococcal serotype or not.

Unfortunately, the false-positive information that this culture-independent method introduced is difficult to quantitate. While real time PCR-serotyping is predicted to add somewhat more specificity for pneumococcal targets than cmPCR, real time PCR may similarly detect non-pneumooccal strains among carriage specimens. For example, we applied our recently developed triplexed real time assay (Pimenta et al., 2012) on the non-pneumococcal strains depicted in Fig. 1 that were cmPCR-positive. While we found that the *S. oralis* strains with the cmPCR subtypes 376.12F and 291.33F were each strongly positive for the corresponding real-time PCR assay in triplex or monoplex format (for detecting 12F/12A/44/46 and 33F/33A/37 respectively), the other 7 strains were uniformly negative for their respective real time PCR assay. It is possible that any single PCR assay used for detection of pneumococci in the upper respiratory tract has a risk of cross-reaction with related mitis group streptococci. We have this concern for the CDC *lytA* assay, however, we presently have no data suggesting that it cross-reacts with non-pneumococcal species. Currently we can only state from the data shown in Table 1 that while we found *lytA*-positivity for numerous specimens from which we did not recover pneumococci, especially from adult NP/OPS, we did not encounter any *lytA*-negative specimens that yielded pneumococcal isolates. The nine cmPCR-positive non-pneumococcal strains described in this study were found to be *lytA*-negative. Although this finding is not conclusive, it is consistent with observations that indicate the specificity of the CDC *lytA* assay for pneumococcal identification (Carvalho et al., 2007).

The majority of these non-pneumococcal homolog sequence subtypes have not been documented or characterized at this time. Indeed, except for the cmPCR type 10F/10C/33C subtypes which are highly homologous to known mitis group counterparts (Yoshida et al., 2008; Yang et al., 2009), all of the subtype sequences depicted in Table 8 most closely matched their known pneumococcal counterparts. Similar known *S. oralis* amplicon sequences lie within operons quite similar to their pneumococcal sg10 *cps* operons and encode the apparatus responsible for synthesis of coaggregation receptor polysaccharides (Yoshida et al., 2008; Yang et al., 2009). The limited sequence-based associations made here in no way preclude identical amplicon subtypes from being shared between pneumococci and other related species. On the contrary, such findings are entirely expected, and a case in point is a suspected non-pneumococcal source for the single st39.39 subtype found within a *lytA*-negative specimen (Table 6). While all colony types were screened for non-pneumococcal sources of cmPCR amplicons, only pneumococci and other alpha-hemolytic mitis group species were implicated as cmPCR-positive.

One issue that concerned us was the possibility that the real time *lytA* PCR assay might cross-react with non-pneumococcal species present in the upper respiratory tract. This was especially concerning in view of the relatively high frequency of *lytA*-positive A-NP/OPs relative to pneumococcal culture-positive A-NP/OPs (approximately 2-fold and 5-fold more *lytA*-positives relative to culture-positives in HIV-positive and HIV-negative, respectively, as shown in Table 1). In part this discrepancy could be due to greater technical difficulty in isolating pneumococci from oropharyngeal flora relative to nasopharyngeal flora, as shown by our relatively poor isolation rates from retrospectively tested OP specimens (Table 8). From the limited re-testing results within our laboratories, it appears that we have under-estimated pneumococcal carriage within the A-NP/OPs described here (Tables 8A and 8B). In the cross-comparison of adult NP and OP specimen testing results, we could project a total of 17 more culture-positives among the 70 HIV-positive culture-negative combined NP/OP specimens, which would have resulted in a 55.1% carriage frequency (rather than 40.7% as shown in Table 1). Similarly, among the HIV-negative specimens we missed 5 positive results (4 NPs and 1 OP) corresponding to 28 A-NP/OP specimens that were originally found to be culture-negative. This would translate to 6 additional positives among the original 35 culture-negative NP/OPs (Table 1), more than doubling our original culture-based findings to 27.5%.

While the magnitude of putative non-pneumococcal cmPCR-positive results were evident within the A-NP/OPs (Tables 3 and 4), we demonstrated that the majority of this confounding data was conferred from A-OP specimens (Tables 6 and 7).While we believe that the majority of the cmPCR data shown in Table 2 from C-NPs accurately predicts pneumococci of corresponding sequence types, at least a small percentage of non-pneumococcal cmPCR –positive results can be found within the pediatric nasopharyngeal reservoir, since we isolated a *S. mitis* strain of cmPCR subtype 257.18 from one C-NP specimen.We quantitatively investigated the cmPCR-18C/A/B/F data from C-NPs and found that of the 4 amplicons not corresponding to st18C or st18A pneumococcal isolates, 2 amplicons shared an identical sequence with the published st18A reference (http://www.cdc.gov/ncidod/biotech/files/pcr-oligonucleotide-primers.pdf), one shared sequence identity with the published st18C reference, and one was divergent (257.18) from *lytA*-negative specimen from which the causal *S. mitis* was recovered. Subtype 32.10 is also likely to be present within non-pneumococcal pediatric nasopharyngeal flora, since it was originally identified from *S. oralis* reference strains (Carvalho et al., 2012) and was also associated with a single *lytA*-negative C-NP (Table 8). In contrast to cmPCR type 18C/A/B/F, several other cmPCR types corresponding to important serotypes included in conjugate vaccines (cmPCR types 1, 19A, 19F, and 23F) were reflected by single sequence subtypes among multiple sequenced amplicons, were associated only with pneumococcal isolates, and were not found among *lytA*-negative specimens in this study (Table 6). These results indicate that these particular cmPCR reactions are potentially pneumococcal-specific.

Although the nasopharynx is believed to be the principal carriage reservoir of *S. pneumoniae* in children, the organism also resides in the oropharynx. Although NP sampling is believed to be more representative overall of carriage strains than OP sampling, using both NP and OP sampling in adults modestly enhanced the detection of pneumococcal carriage (Watts et al., 2004). In contrast, a large-scale study performed in Burkina Faso indicated that adding OP swab data to NP swab data increased culture-based carriage detection by 60% (Mueller et al., 2012). While our small cross-comparison of 56 NP, OP, and combined NP/OP specimens indicate that NP specimens were the preferred specimen for pneumococcal isolation, it is important to note that our methods differ in that we employ broth-preculture before plating for isolation. Additionally we do not employ gentamycin selection in our isolation plates, however, we have found that using this selection does not improve our results in recovering pneumococci from NP or OP specimens (data not shown).

In conclusion, while usage of culture-independent cmPCR for more sensitive detection of pneumococcal serotypes in broth-enriched NP specimens appears promising, more analysis is necessary. Presently it is our opinion that pneumococcal strain isolation is a necessary component of carriage studies, and that serotyping by conventional or molecular methods should be done on colonies confirmed to be pneumococcal. When performing cmPCR to assess pneumococcal serotype distribution on specimens directly, it is necessary to correlate specific “cmPCR-serotype” amplicon sequence subtypes with their existence in pneumococcal strains of the concordant serotype. We admit that even this precaution is not completely satisfactory, due to the possible presence of specific amplicon sequence subtypes in both pneumococcal and non-pneumococcal strains. Enrichment culture to enhance pneumococcal recovery and detection that combines NP specimens together with OP specimens should be avoided, since OP specimens are apparently a much richer source of non-pneumococcal mitis group strains that confound cmPCR serotype assessment and potentially mask the presence of pneumococcal strains.

## Supplemental Information

10.7717/peerj.97/supp-1 Table S1 New cmPCR amplicon sequence subtypes of less than 200 bp in length.

## Additional Information and Declarations

### DNA Deposition

The following information was supplied regarding the deposition of DNA sequences: GenBank: KC771356–KC771416, KC808157–KC808165 and KC779228–KC779254.
