# Supplementary material for: Non-pneumococcal mitis-group streptococci confound detection of pneumococcal capsular serotype-specific loci in upper respiratory tract
Source: PeerJ. 2013 Jun 25;1:e97. doi: 10.7717/peerj.97 (PMC3698467; doi:10.7717/peerj.97)
Supplement: Table S1 [file peerj-01-97-s001.docx]

Supplementary Table 1. New cmPCR amplicon sequence subtypes of less than 200 bp in length found during this work referred to in Table 6.

cmPCR type 6AB 191 bp *wciP* fragment from unknown species

>subtype141.6

GGGTGTACGGCAGGCTTTAATCATGCATTGTTAGAGATGGTTCCTTCAGTTGATATTGAT

AAAGATTATTTATATATAGAAAAACTGTCTCATGATAATTATTTTGCAAAGTTTGCACTA

GAGTATGGGAAGGTGTTGTTCTGCCCTGAGCAACTGGTCTTGTATCGAAGACACGGACAT

AATGTAACAAC

cmPCR type 6AB 191 bp *wciP* fragment from serotype 6B *Streptococcus pneumoniae*

>subtype 359.6

GGGTGTACTGCAGGTTTTAATCATGCATTGCTAGAGATGGTTCCTTCAGTTGATATTGAT

AAAGATTATTTATATATAGAAAAACTGTCTCATGATAATTATTTTGCAAAGTTTGCACTA

GAGTATGGGAAGGTGTTGTTCTGCCCTGAGCAACTGGTCTTGTATCGAAGACATGGACAT

AATGTAACAAC

cmPCR type 6AB 191 bp *wciP* fragment from unknown species

>subtype 267.6

GGGTGTACTGCAGGTTTTAATCATGCATTGCTAGAGATGGTTCCTTCAGTTGATATTGAT

AAAGATTATTTATATATAGAAAAACTGGCTCATGATAGTTATTTTGCAAAGTTTGCACTA

GAGTATGGGAAGGTGTTGTTCTGCCCTGAGCAACTGGTCTTGTATCGAAGACACGGACAT

AATGTAACAAC

cmPCR type 10F/10C/33C 192 bp *wzx* gene fragment from unknown species.

>subtype 49.10

TAGAATATGCTAGACATCATTTAAAGCCGGTCATATTATTATTCCTTCCTCAAGTAGCTA

TTTCTTTATACATTACGCTGGATCGTACCATGCTTGGAGCCTTAGCTTCTACAAAAGATG

TAGGGATTTATGACCAGGCCCTAAAATTAGTAAATATCCTTCTGACCTTGGTAACTTCCT

TGGGAAGCGTTA

cmPCR type 10F/10C/33C 192 bp *wzx* gene fragment from unknown species.

>subtype 32.10

TAGAATATGCTAAGCATCATTTAAAGCCGGTCATATTATTATTCCTTCCTCAAGTAGCTA

TTTCTTTGTACATTACGCTGGATCGTACCATGCTTGGAGCCTTAGCTTCTACAAAAGATG

TAGGAATTTATGACCAGGCCCTAAAATTAGTAAATATCCTTCTGACCTTGGTAACTTCCT

TGGGAAGCGTTA

cmPCR type 10F/10C/33C 192 bp *wzx* gene fragment from unknown species.

>subtype 300.10

TAGAATATGCTAGACATCATTTAAAGCCGGTCATATTATTATTCCTTCCTCAAGTAGCTA

TTTCTTTGTACATTACGCTGGATCGTACCATGCTTGGAGCCTTAGCTTCTACAAAAGATG

TAGGAATTTATGACCAGGCTCTAAAATTGGTAAATATCCTTCTGACCTTGGTAACTTCCT

TGGGAAGCGTTA

cmPCR type 10F/10C/33C 192 bp *wzx* gene fragment from unknown species.

>subtype 248.10

TAGAATATGCTAGACATCATTTAAAGCCGGTCATATTATTATTCCTTCCTCAAGTAGCTA

TTTCTTTGTACATTACGCTGGATCGTACCATGCTTGGAGCCTTAGCTTCTACAAAAGATG

TAGGGATTTATGACCAGGCCCTAAAATTAGTAAATATCCTTCTGACCTTGGTAACTTCCT

TGGGAAGCGTTA

cmPCR type 10F/10C/33C 192 bp *wzx* gene fragment from unknown species.

>subtype 265.10

TAGAATATGCTAGACATCATTTAAAGCCGGTCATATTATTATTCCTTCCTCAAGTAGCGA

TTTCTTTGTACATTACGCTGGATCGTACCATGCTTGGAGCCTTAGCTTCTACAAAAGATG

TAGGGATTTATGACCAGGCCCTAAAATTAGTAAATATCCTTCTGACCTTGGTAACTTCCT

TGGGAAGCGTTA

cmPCR type 10F/10C/33C 192 bp *wzx* gene fragment from *Streptococcus oralis* strain 6024.

>subtype 378.10

TAGAATTTGCTAGGTATCATTTAAAGCCAGTCATATTATTATTCCTTCCTCAAGTAGCTA

TTTCTTTGTACATTACGCTGGATCGTACCATGCTTGGAGCCTTAGCTTCTACAAAAGATG

TAGGGATTTATGACCAGGCCTTAAAATTAGTAAATATCCTTCTGACCTTGGTAACTTCCT

TGGGAAGCGTTA

cmPCR type 10F/10C/33C 192 bp *wzx* gene fragment from *Streptococcus parasanguinis* strain 6121.

>subtype 387.10

TAGAATATGCTAGGCATCATTTAAAGCCGGTCATATTATTATTCCTTCCTCAAGTAGCTA

TTTCTTTGTATATTACGCTGGATCGTACCATGCTTGGAGCCTTAGCTTCTACAAAAGATG

TAGGAATTTATGACCAGGCTCTAAAGTTGGTAAATATCCTTCTGACCTTGGTAACTTCCT

TGGGAAGCGTTA
